# The impact of selfies on body image satisfaction and the chain mediating role of self-objectification and narcissistic personality

**DOI:** 10.3389/fpsyg.2023.1292708

**Published:** 2024-01-05

**Authors:** Yaqi Mei, Wenyu Yang, Can Wang

**Affiliations:** School of Nursing, Anhui Medical University, Hefei, China

**Keywords:** selfies, body image satisfaction, self-objectification, narcissistic personality, college student

## Abstract

This study explores the mediating effect of self-objectification and narcissistic personality on the relationship between selfies and body image satisfaction. A total of 368 college students were administered a survey that included general information, selfie-related questions, a body image satisfaction scale, a body surveillance scale, and a narcissistic personality scale. Selfies, body image satisfaction, self- objectifica- tion, and narcissistic personality were shown to be positively correlated. Mediation modeling analysis found that selfies had a direct effect on body image satisfaction and that self-objectification and narcissistic personality mediated this relationship. Consequently, this study holds suggestions for researchers and educators searching for better exploration and attention to improve the content of education, guide students to set up a correct moral outlook, outlook on life and values.

## Introduction

### Background

The rapid development of internet information technology has made social network- ing an important method of communication in daily life. According to the 2021 Statistical Report on Internet Development in China, there are 1.032 billion internet users in China, representing a penetration rate of 73%, and an average of 28.5 h a week is spent online (China Internet Network Information Center, 2021). Social networking sites such as QQ, WeChat, Weibo, and Douyin are becoming increasingly popular. The most common uses of these sites include posting photos, statuses, logs, and comments. On college student sites, selfies are particularly important content for self-presentation. These photos also impact other aspects of student life, heighten one’s self-awareness and impacts one’s self-concept (Megan and David, 2021). The act of taking a selfie typically involves enabling the front-facing feature on a smartphone to photograph oneself (Megan and David, 2021), giving the individual great control over the process (Sarah and Lara, 2017). Studies indicate that most college students take selfies, and more than half alter or embellish them before posting (Chen, 2018). Selfies are posted to gain attention or recognition and promote interaction through tags and comments. Self-perception theory suggests that when individuals present certain aspects of themselves to others, they become more prominent and are more likely to guide future behavior. In other words, one’s own attitude or behavior is determined by the behavior of others (Bem, 1972). This theory tells that college students’ perception of body image changes with their viewers’ ratings after they post images on social media platforms, which may have positive or negative effects. The development and use of beautification functions, such as filters that remove blemishes, add makeup and remodel the face, has encouraged more young people to focus on their appearance (Chen et al., 2019; Shome et al., 2020).

Body satisfaction is defined as self-perception of an individual’s body image (Peter and Valkenburg, 2014) and is heavily influenced by (and in) society through self-perception, attitudes about others, and interpersonal communication (Angelova and Utermohlen, 2013). Previous studies show that selfie comparisonsand negative comments on social networking sites increase worry, tension, and uneasiness that can promote body image dissatisfaction (Fardouly et al., 2015; Kim and Chock, 2015). Findings indicate that 58.57% college students are un- happy with their body image (Li and Wu, 2016) and this is closely associated with self-esteem, depression, anxiety, and psychological health and eating disor-ders (Wiseman et al., 2005; Derenne and Beresin, 2006; Choi and Choi, 2016; Peng et al., 2017; Gerada, 2020). Body dissatisfaction is strongly associated with the mentalhealth of individuals, especially young people (Littleton and Ollendick, 2003). Thus, more research is focusing on the relationship between selfies and body satisfaction. Self-objectification is a common phenomenon in the current era of social network interactions. This objectifying gaze is internalized by posting selfies, causing people to adopt an observer’s perspective on their own body or to objectify themself (Fredrickson and Roberts, 1997). According to the objectification theory, individual experience objectification will lead to the improvement of individual self objectification. Thus, individuals with high self-objectification will focus on external evaluations but ignore internal qualities such as like ability and morality (Fredrickson and Roberts, 1997). The focus of objectification theory is appearance, one-sided self-image recognition, and the psychological process of examining and evaluating one’s own body as an object. While college students are in an important stage of value building, positive self-recognition and comprehensive self-evaluation are particularly important. Studies indicate that social networks are a common way for individuals to experience sexual objectification (Sun et al., 2013) and lead to the development of negative emotions (Abbie and Moin, 2021). Thus, self-objectification may play an inter-mediary role in the relationship between selfies and body image satisfaction onsocial networks.

One study found that posting selfies on social network sites is linked to narcissism (Yang et al., 2017; Bai et al., 2020). Social networking site use prompts individuals to continually internalize the positive self-image they portray and facilitates the receipt of supportive feedback from others, which in turn enhances the individual’s level of narcissism (Gentile et al., 2012; Halpern et al., 2016). High narcissistic individuals are more likely to engage in positive self-presentation in social networks like selfies (Jiang and Jin, 2018). Indeed, media selection theory emphasizes that individual behavioral traits are affected by media (Trepte and Reinecke, 2013). One of these traits, narcissistic personality, describes the structure of behavior patterns in an individual’s life. According to Koh (1996), a narcissistic personality is associated with the follo-wing qualities: internal control, a need for achievement, a moderate level of risk-taking, innovation, high self-confidence, and a high tolerance for ambiguity. As an individual posts more selfies on social network sites, they are encouraged to receive and internalize supportive feedback from others and their level of narcissism increases (Halpern et al., 2016). An excessive focus on appearance and physical attractiveness is a hallmark of narcissism (Vazire et al., 2008). Highly narcissistic individuals are provided the opportunity to comment on, pay attention to, and be recognized by selfies on social network sites, which direct-ly impacts their body image satisfaction. Thus, it can be inferred that having a narcissistic personality mediates the relationship between selfies and body image satisfaction.

### Research purpose

The present study sought to clarify the nature of the relationship between selfies and body image satisfaction and to test the mediating effect of self-objectification and narcissistic personality on this relationship among college students. The study also explored how the two forms of narcissism, explicit and implicit (Pincus et al., 2009) impact this relationship.

## Methods

### Participants and procedures

This was a cross-sectional study that used an anonymous self-administered question- naire. A total of 436 college students from Anhui Medical University were invited to participate in the survey between March and June 2022. Prior to launch, the purpose of the study was explained to the participants with an emphasis that the data were collected for scientific research purposes only and that personal privacy would be strictly protected to eliminate participant concerns and ensure data quality. A total of 368 survey responses were obtained for a response rate of 84.40%. Respondents were 78.5% female with an average age of 19.31 ± 1.13 years.

### Measurements of variables

#### Selfies

A 27-item self-administered instrument developed by a Chinese scholar (Wu, 2015) was used for the selfie scale, with sample items such as “Selfie can let me know more friends.” It comprises eight dimensions: behavior (2 items), perceived usefulness (3 items), perceived ease of use (3 items), use attitude (5 items), intention to use (4 items), satisfaction (3 items), motivation (3 items) and human need (4 items). All items use a seven-point Likert scale ranging from 1 (completely disagree) to 7 (completely agree). The coefficient alpha for this scale in the study was 0.967.

#### Body image satisfaction

A 9-item self-administered instrument developed by Cash et al. (2002) was used to measure body image satisfaction, with sample items such as “How satisfied are you with your appearance right now.” All items use a nine-point Likert scale ranging from 1 (completely disagree) to 9 (completely agree). The coefficient alpha for this scale in the study was 0.954.

#### Self-objectification

The Body Surveillance Scale developed by Hyde and Mckinley (2006) and revised by Chen and Jiang (2007) was used to quantify self-objectification, with sample items such as “I do not pay much attention to how I look.” This scale includes 8 items and is scored on a seven-point scale ranging from 1 (completely disagree) to 7 (completely agree). The coefficient alpha for this scale in the study was 0.761.

#### Narcissistic personality

A 28-item self-administered instrument developed by Zhen and Huang (2005) was used to measure narcissistic personality, with sample items such as “People often disappoint me.” This includes two subscales, the Explicit Narcissism Scale which includes a sense of privilege, superiority, sense of lust, and self-admiration, and the Implicit Narcissism Scale which includes sensitivity, sense of privilege, and self- admiration. All items use a five-point Likert scale from 1 (completely disagree) to 5 (completely agree). The coefficient alpha for the two subscales in the study were 0.912 and 0.885, respectively.

#### Demographics

The study also collected data on participant demographics, including gender and age.

### Statistical analysis

Common method bias, descriptive, Pearson correlation, and PROCESS analyses were conducted using SPSS 21.0. Common method bias included all dimensions of the four variables. Descriptive statistics included frequency, percentage, mean and standard deviation. Pearson correlation analysis assessed the relationship between the overall scores of the four variables. PROCESS analysis tested the chain mediating role of self-objectification and narcissistic personality on the relationship between selfies and body image satisfaction. In all these analyses, a two-tailed probability of <0.05 was considered statistically significant.

## Results

All data conforms to a normalized distribution (*p* > 0.05).

### Common method bias

Harman’s single-factor test was performed to test for common method bias (Podsakoff and Organ, 1986). The results met the Common method bias standard proposed by Hair et al. (1998). There were four factors extracted with feature values >1. The explanatory variance of the first factor was 28.659%, which was lower than the reference value is 40%. Thus, it was concluded that no common method bias existed in the data.

### Descriptive statistics and correlation analysis

Scores on the selfie, body image satisfaction, self-objectification, explicit narcissism, and implicit narcissism scales were 104.98 ± 23.28, 33.08 ± 8.99, 35.49 ± 6.76, 59.25 ± 11.16, and 43.13 ± 8.84, respectively. According to Table 1, a significant positive correlation was found between all the dimensions (Table 1).

**Table 1:** Correlation analysis of the variables.

	1	2	3	4	5
1. Selfies	1				
2. Body image satisfaction	0.325***	1			
3. Self-objectification	0.147**	0.439***	1		
4. Explicit narcissism	0.245***	0.313***	0.305***	1	
5. Implicit narcissism	0.088*	0.140**	0.163**	0.826***	1

*p < 0.05, **p < 0.01, ***p < 0.001.

### Mediation effect test

This study examined the mediating effect of the participants’ self-objectification and explicit or implicit narcissism on the relationship between selfies and body image satisfaction based on the premise that gender and age were controlled.

### The mediating role of self-objectification and explicit narcissism between selfies and body image satisfaction

**Figure 1:**
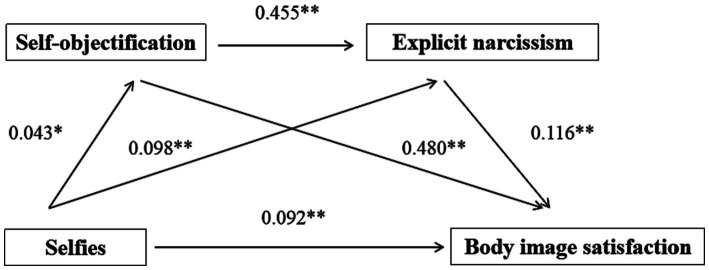
The chain mediating effect of self-objectification and explicit narcissism.

Selfies significantly and positively predicted self-objectification (*β* = 0.043, *p* < 0.05), explicit narcissism (*β* = 0.098, *p* < 0.01) and body image satisfaction (*β* = 0.092, *p* < 0.01) (Table 2). Self-objectification significantly and positively predicted explicit narcissism (*β* = 0.455, *p* < 0.01) and body image satisfaction (*β* = 0.480, *p* < 0.001). Explicit narcissism significantly and positively predicted body image satisfaction (*β* = 0.116, *p* < 0.05). Bootstrap test results found that the independent mediating effect of self-objectification and explicit narcissism, and the chain mediating effect of self-objectification-explicit narcissism were significant. The total indirect effect accounted for 26.98% of the total effect (Figure 1 and Table 3).

**Table 2:** Regression analysis of the mediating role of self-objectification and explicit narcissism on the relationship between selfies and body image satisfaction.

Dependent variable	Independent variable	*R*	*R^2^*	*F*	*B*	*SE*	*β*	*t*
Self-objectification	Selfies	0.147	0.022	4.687*	0.043	0.020	0.041	2.165*
Explicit narcissism	Selfies	0.367	0.134	14.645***	0.098	0.033	0.094	2.967**
	Self-objectification				0.455	0.116	0.386	3.925**
Body image satisfaction	Selfies	0.530	0.281	37.095***	0.092	0.023	0.031	3.967**
	Explicit narcissism				0.116	0.0513	0.025	2.267*
	Self-objectification				0.480	0.089	0.161	5.423***

*p < 0.05, **p < 0.01, ***p < 0.001.

**Table 3:** Mediating effect of self-objectification and explicit narcissism.

Path	Effect	95%CI	
		LLCI	ULCI
Selfies-Self-objectification-Body image satisfaction	0.021	0.003	0.043
Selfies-Self-objectification-Explicit narcissism-Body image satisfaction	0.002	0.001	0.007
Selfies-Explicit narcissism-Body image satisfaction	0.011	0.003	0.027
Indirect effect	0.034	0.012	0.061
Direct effect	0.092	0.046	0.137
Total effect	0.126		

### The mediating role of self-objectification and implicit narcissism between selfies and body image satisfaction

**Figure 2:**
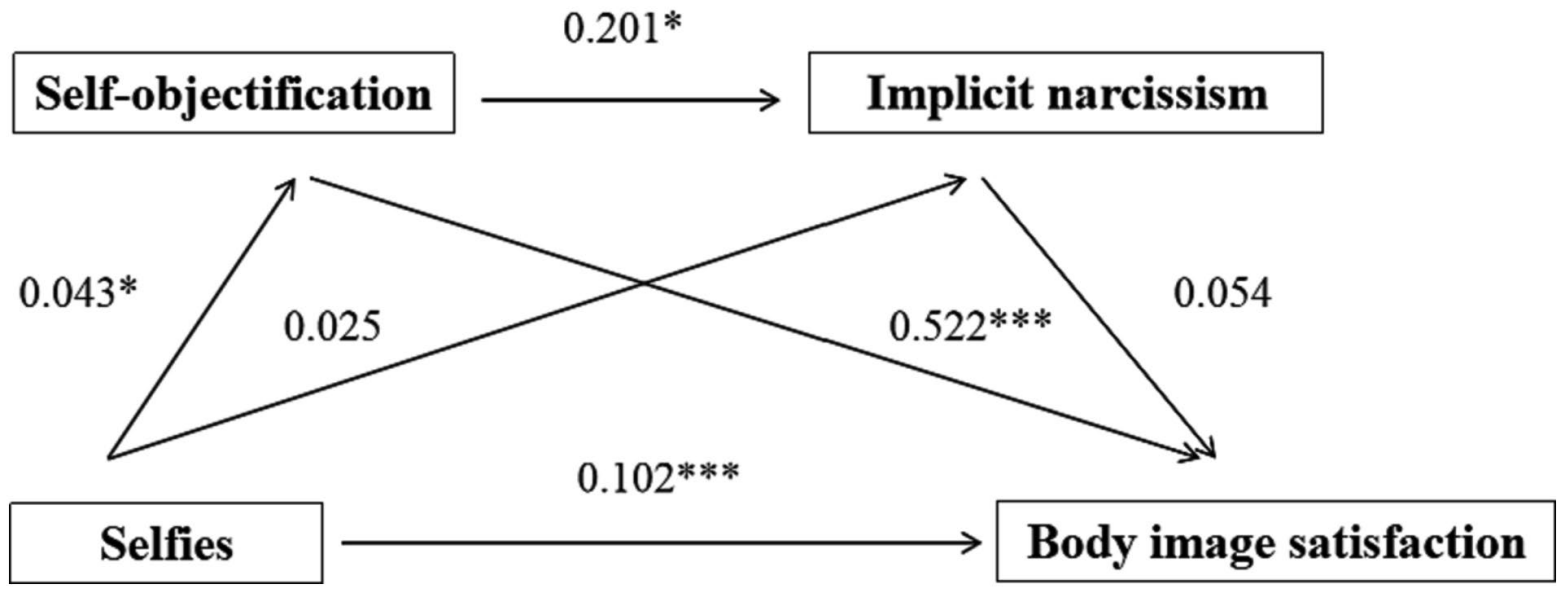
The chain mediating effect of self-objectification and implicit narcissism.

Selfies significantly and positively predicted self-objectification (*β* = 0.043, *p* < 0.05) and body image satisfaction (*β* = 0.102, *p* < 0.001), but had no predictive effect on implicit narcissism (*β* = 0.025, *p* > 0.05) (Table 4). Self-objectification significantly and positively predicted implicit narcissism (*β* = 0.201, *p* < 0.05) and body image satisfac- tion (*β* = 0.522, *p* < 0.001). Implicit narcissism had no predictive effect on body image satisfaction (*β* = 0.054, *p* > 0.05). According to bootstrap test results, the independent mediating effect of self-objectification was significant, but the independent mediating effect of implicit narcissism and the chain mediating effect of self-objectification- implicit narcissism was not. The total indirect effect accounted for 19.05% of the total effect (Figure 2 and Table 5).

**Table 4:** Regression analysis of the mediating role of self-objectification and implicit narcissism on the relationship between selfies and body image satisfaction.

Dependent variable	Independent variable	*R*	*R^2^*	*F*	*B*	*SE*	*β*	*t*
Self-objectification	Selfies	0.147	0.022	4.687*	0.043	0.020	0.041	2.165*
Implicit narcissism	Selfies	0.176	0.031	2.813	0.025	0.027	0.067	0.914
	Self-objectification				0.201	0.098	0.289	2.060*
Body image satisfaction	Selfies	0.515	0.266	31.372***	0.102	0.023	0.035	4.445***
	Implicit narcissism				0.054	0.061	0.041	0.881
	Self-objectification				0.522	0.087	0.159	6.014***

*p < 0.05, ***p < 0.001.

**Table 5:** The mediating effect of self-objectification and implicit narcissism.

Path	Effect	95%CI	
		LLCI	ULCI
Selfies-Self-objectification-Body image satisfaction	0.022	0.003	0.045
Selfies-Self-objectification-Implicit narcissism-Body image satisfaction	0.001	−0.001	0.003
Selfies-Implicit narcissism-Body image satisfaction	0.001	−0.001	0.011
Indirect effect	0.024	0.003	0.047
Direct effect	0.102	0.057	0.147
Total effect	0.126		

## Discussion

Consistent with prior findings, this study found that selfies directly predicted body image satisfaction and self-objectification (Chen, 2018; Duan et al., 2020). College students post selfies on social networking sites such as Wechat and Weibo after beautifying their faces and gaining likes and comments from viewers. This way of displaying appearance satisfies an individual’s need for attention and the positive emotional experience gained in this manner provides self-affirmation and improves body image satisfaction. When individuals take selfies or post photos, they tend to focus on their appearance rather than their internal attributes. The use of social networks further strengthen the individual’s emphasis on appearance and ideal body image and helps to solidify self-objectification.

The current findings also suggest that selfies significantly and positively predict explicit narcissism but have no predictive effect on implicit narcissism. Individuals with explicit narcissism lack self-awareness and like to highlight themselves to solicit attention and praise from others, while those with implicit narcissism tend to reduce communication with others and hope that others will acknowledge their own value. They have a fragile sense of self-worth and while they do not like to show themselves off, they long for attention (Pincus et al., 2009). Studies indicate that most selfies on social networks are posted by young women (Ding et al., 2016; Tiggemann and Velissaris, 2020). Indeed, 78.5% of the subjects in this study were female college students, which validates views on gender differences and same-sex competition explained by evolutionary psychology. While men pay more attention to physical appearance, women are more focused on inner abilities and personality (Su and Ye, 2021). To increase their chances of attention and courtship, women have evolved ways of gaining male attention through physical attraction (Li et al., 2011). One of these methods is by showing themselves off by posting selfies.

The current study also found that self-objectification has a mediating role in the relationship between selfies and body image satisfaction. The findings indicate that the self-portraits of college students on social networking sites can affect body image satisfaction through self-objectification. Young people, especially college students, have become the leaders of information communication through social networks and are often particularly careful about how their appearance is presented. Frequent online selfie display and visitors attention further strengthen an individual’s attention to appearance, body image ideals, and body image, thereby affecting body image satisfaction.

The findings shown here illustrate that explicit narcissism has a clear mediating role between selfies and body image satisfaction. The selfies college students post on social networking sites appear to affect their body image satisfaction through explicit narcissism. High-level narcissists tend to have more frequent social network status updates, longer time social network posts, and more web visitors (Walters and Horton, 2015; Liu and Baumeister, 2016; Moon et al., 2016). Explicit narcissists are particularly expressive, devoting time and energy to expressive social networking activities, including selfies. Through this positive appearance display and positive interaction with others, it is beneficial to build a good self-image, so as to improve their body satisfaction.

This study identified a chain mediating role of self-objectification and narciss- istic personality on the relationship between selfies and body image satisfaction. College students who like to post selfies on social networking sites tend to pay more attention to their external characteristics, and comments from others directly impact their level of self-objectification. The more positive their self-image and the more praise they receive on social media, the higher their level of narcissism (especially the explicit form), and the greater impact this has on self-image satisfaction.

### Practical implications

Firstly, higher education educators should correctly guide college students to view the impact of social networking site selfies on individuals comprehensive-ly and objectively. Reduce the risk of body image dissatisfaction by encouragi-ng social networking site users to give more positive comments and fewer negative comments to others’ selfies. Secondly, through mental health education to help college students establish correct values, reduce the importance of physical appearance in college students’ sense of self-worth, and help them position their self-worth in areas such as knowledge and ability, thus reducing the degree of self-objectification and narcissistic personality among college students. Finally, correctly guide college students to appreciate their bodies, accept body imperfections, and enhance body satisfaction.

### Limitations and future research directions

This study only involved college students from one university in Anhui Provin-ce, so the findings may not be generalizable to other populations. Investigating the experiences of college students from other types of schools will increase the value of the research findings. In addition, all variables in this study were assessed on a self-assessment scale and based on a cross-sectional study, which has the shortcoming of participants’ subjective factors influencing the assessme-nt process. Future studies can adopt multiple time points and perspectives for data collection, such as collecting data in two or three phases.

## Data availability statement

The original contributions presented in the study are included in the article/supplementary material, further inquiries can be directed to the corresponding author.

## Ethics statement

The studies involving human participants were reviewed and approved by Anhui Medical University. The participants provided their written informed consent to participate in this study. Written informed consent was also obtained for the publication of any potentially identifiable images or data included in this article.

## Author contributions

YM: Data curation, Funding acquisition, Writing – original draft, Writing – review & editing. WY: Data curation, Investigation, Writing – review & editing. CW: Data curation, Investigation, Writing – review & editing.
